# Role of the ERK1/2 Signaling Pathway in the Replication of Junín and Tacaribe Viruses

**DOI:** 10.3390/v10040199

**Published:** 2018-04-17

**Authors:** Jesús E. Brunetti, Sabrina Foscaldi, Verónica M. Quintana, Luis A. Scolaro, Nora López, Viviana Castilla

**Affiliations:** 1 Laboratorio de Virología, Departamento de Química Biológica, Facultad de Ciencias Exactas y Naturales, Universidad de Buenos Aires, Buenos Aires C1428EGA, Argentina; jebrunetti@qb.fcen.uba.ar (J.E.B.); vmquintana@qb.fcen.uba.ar (V.M.Q.); luisco@qb.fcen.uba.ar (L.A.S.); 2 Centro de Virología Animal (CEVAN), Consejo Nacional de Investigaciones Científicas y Técnicas (CONICET), Buenos Aires C1440FFX, Argentina; fosqui81@hotmail.com (S.F.); nlopezcevan@centromilstein.org.ar (N.L.)

**Keywords:** Junín virus, Tacaribe virus, arenavirus, ERK, cell signaling, replication

## Abstract

We have previously shown that the infection of cell cultures with the arenaviruses Junín (JUNV), Tacaribe (TCRV), and Pichindé promotes the phosphorylation of mitogen-activated protein kinases (MAPKs) extracellular signal-regulated kinases 1 and 2 (ERK1/2) and that this activation is required for the achievement of a productive infection. Here we examined the contribution of ERK1/2 in early steps of JUNV and TCRV multiplication. JUNV adsorption, internalization, and uncoating were not affected by treatment of cultured cells with U0126, an inhibitor of the ERK1/2 signaling pathway. In contrast, U0126 caused a marked reduction in viral protein expression and RNA synthesis, while JUNV RNA synthesis was significantly augmented in the presence of an activator of the ERK1/2 pathway. Moreover, U0126 impaired the expression of a reporter gene in a TCRV-based replicon system, confirming the ability of the compound to hinder arenavirus macromolecular synthesis. By using a cell-based assay, we determined that the inhibitor did not affect the translation of a synthetic TCRV-like mRNA. No changes in the phosphorylation pattern of the translation factor eIF2α were found in U0126-treated cells. Our results indicate that U0126 impairs viral RNA synthesis, thereby leading to a subsequent reduction in viral protein expression. Thus, we conclude that ERK1/2 signaling activation is required for an efficient arenavirus RNA synthesis.

## 1. Introduction

Arenaviruses are enveloped RNA viruses containing a bisegmented single-stranded genome with ambisense coding strategy. The L segment codes for the RNA-dependent RNA polymerase (L) and the small matrix protein (Z), while the S segment encodes the nucleocapsid protein (N) and a glycoprotein precursor (GPC). Some members of this family cause hemorrhagic fever disease in humans and represent an important public health concern in their endemic regions, such as Lassa and Lujo viruses in Africa, Sabiá virus in Brazil, Guanarito virus in Venezuela, Chaparé and Machupo viruses (MACV) in Bolivia, and Junín virus (JUNV) in Argentina [1,2]. JUNV is the etiological agent of Argentine hemorrhagic fever (AHF), with the administration of immune plasma being the only therapeutic intervention against the disease. Alternative antiviral therapies are needed due to troubles in maintaining adequate stocks of plasma and the absence of effective interventions for treated patients that progress to a neurologic-hemorrhagic phase [3,4]. The development and implementation of a vaccination program within the endemic area, based on the live attenuated Candid #1 vaccine, has markedly reduced the incidence of AHF. Nevertheless, Candid #1 is not recommended for children and pregnant women and although this vaccine showed partial cross-protection for the arenavirus MACV, it has only been approved in Argentina [5].

It is well established that viruses manipulate cell signaling machinery for their own benefit. Mitogen-activated protein kinases (MAPKs), families of serine/threonine kinases that respond to extracellular stimuli, transduce signals from the cell membrane to the nucleus, playing important roles in the replication of several RNA and DNA viruses [6,7,8]. The best-known MAPK families are the extracellular signal-regulated kinases 1 and 2 (ERK1/2), associated with growth or proliferation signals and the c-Jun N-terminal kinase (JNK) and p38 pathways, which are involved in the response to environmental and inflammatory stress. The main components of the ERK1/2 cascade are three sequentially acting kinases: Raf, MEK1/2 and ERK1/2. Activated ERK1/2 MAPKs not only translocate to the nucleus where they phosphorylate transcription factors triggering changes in gene expression but also mediate the phosphorylation of several cytoplasmic substrates. In addition, ERK1/2 pathway plays a relevant role in the replication of several viruses and the regulation of innate immune response against viral infections [9,10,11,12,13,14,15,16,17,18,19,20]. Based on that, inhibition of the ERK1/2 pathway has been proposed as a novel antiviral strategy [9,21,22]. 

We have previously reported that JUNV infection induces a biphasic activation of the ERK1/2 pathway, with a clear peak of ERK phosphorylation during the first minutes of infection and a late and sustained ERK activation from 7 h after infection onwards. In addition, we demonstrated that treatment with a MEK inhibitor, the compound U0126, as well as ERK1/2 silencing mediated by small interfering RNAs, caused a strong reduction in the production of JUNV infectious particles in monkey and human cells, indicating that ERK1/2 activation is required for JUNV multiplication. Moreover, ERK1/2 phosphorylation was also detected in cell cultures infected with Tacaribe (TCRV) and Pichindé (PICV) arenaviruses and treatment with U0126 caused a significant inhibition of TCRV and PICV multiplication [23].

Here we investigated the role of the ERK1/2 cascade in arenavirus infection by performing a more detailed analysis of the effect of U0126 on early steps of JUNV and TCRV multiplication.

## 2. Materials and Methods

### 2.1. Cells, Viruses, and Compounds

Vero cells were grown in modified Eagle’s medium (MEM) (Invitrogen; Carlsbad, CA, USA) supplemented with 5% newborn calf serum (NBCS) and gentamicin (50 µg/mL). BSR cells (a clone of BHK-21 cells) were grown using Glasgow minimum essential medium (GMEM; Invitrogen) supplemented with glutamine (2 mM), 10% fetal calf serum (FCS) (Invitrogen), and penicillin (100 U/mL)-streptomycin (100 µg/mL) (Invitrogen). All experiments were performed in serum-free medium. The attenuated strain of JUNV (XJCl3), was handled at biosafety level 2 by trained personnel vaccinated with Candid#1 live attenuated vaccine. JUNV stock was obtained in BHK-21 cells. Viral infectivity was assayed on Vero cells by a plaque formation assay using MEM supplemented with 0.5% NBCS, 0.7% methylcellulose and gentamicin (50 µg/mL). 

U0126 (Cell Signaling Technology; Danvers, MA, USA), ribavirin (Sigma-Aldrich; St. Louis, MI, USA) and phorbol 12-myristate 13-acetate (PMA, Sigma-Aldrich) were prepared in dimethyl sulfoxide (DMSO) and further diluted in serum-free medium before use; the same percentage of DMSO diluted in serum-free medium was used in control untreated cultures.

### 2.2. Antibodies

Mouse anti-JUNV G1 protein monoclonal antibody (mAb) QC03-BF11 [24] was used as primary antibody in immunofluorescence assays. Goat anti-mouse IgG conjugated to fluorescein isothiocyanate (FITC) (Sigma-Aldrich) was used as secondary antibody.

Western blot analysis was performed employing mouse anti JUNV N protein NA05-AG12 mAb [24] and rabbit anti phospho-ERK1/2 (p-ERK) (Cell Signaling), anti ERK1/2 (ERK) (Santa Cruz Biotechnology; Dallas, TX, USA) and anti phospho-eIF2α (p-eIF2α) (Cell Signaling) antibodies. Peroxidase anti-rabbit IgG (Amersham; Little Chalfont, Buckinghamshire, UK) or peroxidase anti-mouse Ig G (Sigma-Aldrich) were used as secondary antibodies.

### 2.3. Plasmids

Plasmids pTCRV N and pTCRV L, expressing TCRV N and L proteins, respectively, were previously constructed [25]. The plasmid pMG-FLUC expresses a minigenome (designated “MG-FLUC”), which is an analog to the TCRV S genomic segment, and comprises the firefly luciferase (FLUC) open reading frame (ORF) in an antisense orientation, flanked by the complete S genome non-coding sequences [26,27,28]. 

Plasmid p5′wt/3′wt_2, which expresses a transcript that mimics the wild-type TCRV N mRNA; plasmid pβGlo/poly(A) expressing a cell-like mRNA (named β-Glo/poly(A)) that bears the 5′ untranslated region (UTR) of the human β-globin mRNA and a poly(A) tract flanking the FLUC ORF, and plasmid pRLUC, used to generate a synthetic mRNA expressing *Renilla reniformis* luciferase enzyme (RLUC), have been previously constructed [29]. 

Plasmid pCMV-T7pol expresses the bacteriophage T7 RNA polymerase under the control of the cytomegalovirus promoter [30] and was kindly provided by Martin A. Billeter (University of Zurich, Irchel, Switzerland).

### 2.4. Viral Adsorption Assay

Vero cells pre-incubated in MEM containing or not U0126 (15 µM) during 30 min were infected at a multiplicity of infection (m.o.i.) of 1 PFU/cell in the presence or absence of U0126 for 1 h at 4 °C. Following extensive washes, cells were lysed with 3 cycles of freezing and thawing, cell debris were removed by low speed centrifugation and viral titers in the supernatants were quantified by plaque assay.

### 2.5. Viral Internalization Assay

Vero cells were pre-treated with MEM containing or not U0126 (15 µM) or chlorpromazine (CZ) (40 µM) for 40 min. Then cultures were infected with approximately 100 PFU of JUNV in the presence or absence of U0126 (15 µM) or CZ (40 µM), for 1 h at 4 °C. After viral adsorption cultures were incubated at 37 °C and at different time points, cells were washed with citric buffer (pH = 3.0) for 1 min to inactivate non-internalized viral particles. Finally, cells were covered with MEM containing methylcellulose and internalized virions were quantified by plaque formation assay.

### 2.6. Viral Uncoating Assay

Vero cells were infected with approximately 100 PFU of JUNV for 1 h at 4 °C and then cultures were transferred at 37 °C. At different time points, U0126 (15 µM) or NH4Cl (50 mM) was added. After 3 h of treatment, cells were washed with citric buffer (pH = 3.0) for 1 min, to eliminate non-internalized virions, and cultures were covered with MEM containing methylcellulose to assess the number of uncoated viral particles by plaque formation assay.

### 2.7. Cell Viability Assay

JUNV infected Vero cells or BSR cells transfected or not with plasmids used for the replicon assay were treated with U0126 and after different times cell viability was determined as the ability of living cells to cleave the tetrazolium salt MTT (3-(4,5-dimethylthiazol-2yl)-2,5-diphenyl tetrazolium bromide) (Sigma-Aldrich).

### 2.8. Western Blot Analysis

JUNV infected Vero cells or BSR cells transfected with plasmids that constitute the TCRV-replicon system, treated or not with U0126 (15 µM), were lysed in sample buffer (Bio Rad; Hercules, CA, USA) and the expression of p-ERK, ERK, p-eIF2α or viral N protein was analyzed by Western blot. SDS-PAGE was performed in 10% polyacrylamide gels and proteins were then transferred to a PVDF membrane (Hybond P; Pharmacia, Amersham, UK). Then membranes were incubated in Tris-buffered saline (TBS) containing 0.1% Tween-20 and 5% bovine serum albumin (blocking buffer) for 2 h at 37 °C, followed by overnight incubation at 4 °C with primary antibodies diluted in blocking buffer. After several washes, membranes were incubated for 1 h at room temperature with secondary antibodies diluted in blocking buffer. Protein bands were visualized by chemiluminescence detection using Western Lightning ECL (PerkinElmer; Waltham, MA, USA) and quantified by using Image J (https://imagej.net/Welcome) for Windows.

### 2.9. Immunofluorescence Assay

For total immunofluorescence assay, cell monolayers were washed three times with phosphate buffer saline (PBS), fixed with paraformaldehyde 4% in PBS for 10 min at room temperature and permeabilized by incubation in PBS containing 0.2% Triton X-100 during 10 min at room temperature. Then, cells were incubated with primary antibodies during 60 min at 37 °C and after three washes with PBS, incubation with secondary antibodies during 60 min at 37 °C was performed. After three washes with PBS cell nuclei were stained with Hoechst 33258 reagent (1 µg/mL) and coverslips were mounted on a 90% glycerin solution in PBS (pH 7.2) containing 2.5% 1,4-diazabicyclo-(2,2,2)-octane (DABCO; Sigma-Aldrich) and visualized in a fluorescence microscope. For membrane immunofluorescence, the procedure was the same with the exception that cell permeabilization with Triton X-100 was omitted. The percentage of infected cells for each sample was obtained by counting at least 300 cells from 20 random selected fields (400× magnification).

### 2.10. Syncytium Formation Assay

U0126 (15 µM) was added to JUNV infected cells at different time points and after 14 h of infection, cells were treated for 30 min at acid pH (MEM pH 5.0) and then incubated in medium at neutral pH. At 24 h post-infection (p.i.) cells were fixed with methanol at room temperature for 15 min and then stained with Giemsa 0.1% in PBS for 15 min. Cells were visualized in an optic microscope and the number and size of syncytia for each sample were calculated by counting at least 300 nuclei from 20 random selected fields (400× magnification).

### 2.11. Quantitative RT-PCR

Total RNA was extracted using TRI reagent (Genbiotech; Antibes, France) and the detection of JUNV genomic S RNA by PCR was performed as previously described [31]. Briefly, cDNA was synthesized using the primer N (+) (5′-CGCACAGTGGATCCTAGGC-3′) complementary to positions 3393 to 3501 of the genomic S RNA. Quantitative PCR reaction was conducted using JUNV-specific primers N (−) (5′-GGCATCCTTCAGAACAT-3′) and N (+), and SYBR Green (Fast Start Universal SYBR Green Master, Roche; Basel, Switzerland), to generate a 186 bp amplification fragment comprising the 3′ end of the N coding sequence. The PCR cycle progression for N (+)/N (−) primers was as follows: 5 min at 95 °C and 45 cycles of 30 s at 95 °C, 20 s at 48 °C, 30 s at 72 °C followed by 10 s at 82 °C using the MyiQ™2 Two Color Real-Time PCR Detection System (BioRad; Hercules, CA, USA). The cellular gene actin was used as standard for normalization. Average viral RNA *C*t values were normalized to the average *C*t values of actin (Δ*C*t = *C*t virus − *C*t actin) and ΔΔ*C*t (Δ*C*t untreated cultures − Δ*C*t drug-treated cultures) based fold change calculations were set using Bio-Rad iQ5 2.1 software. 

### 2.12. Replicon Assay

The TCRV reverse genetic system has been previously described [25,28]. Briefly, BSR cells grown in 24-well microplates were transfected, using Lipofectamine 2000 reagent (Invitrogen), with (amounts per well) 0.5 µg of plasmid pCMV-T7pol, 1.5 µg of pTCRV N, 0.5 µg of pTCRV L and 1.3 µg of plasmid pMG-FLUC. The NP- and L-expressing plasmids were omitted in control cell cultures. RLUC reporter vector pRL-TK (Promega; Madison, WI, USA) (40 ng/well) was added to the transfection mix in order to estimate transfection efficiency. At 4 h post-transfection, supernatants were removed and cells were washed twice with PBS and further incubated in the presence or absence of U0126 (15 µM) for 24 h at 37 °C. Quantification of FLUC and RLUC activities on a GloMax^®^-Multi Detection System (Promega) was performed using Dual-luciferase reporter assay system (Promega). For each sample, FLUC activity was normalized against RLUC activity. Background signal measured in control cell cultures was subtracted from data obtained from cultures transfected with the complete set of plasmids. 

### 2.13. Cell-Based Translation Assay 

Capped synthetic RNAs were obtained by in vitro transcription from T7 promoter-controlled constructs. The virus-like mRNA (5′wt/3′wt_2), mimicking the TCRV N mRNA, comprises the viral 5′UTR fused to the reporter FLUC open reading frame followed by the viral 3′UTR. The cell-like β-Glo/poly(A) transcript bears the 5′UTR from human β-globin, and a 53-nt 3′poly(A) tail flanking the FLUC coding sequence. BSR cells, grown in 24-well plates, were transfected with the indicated synthetic mRNA (100 ng/well) along with the RLUC mRNA (50 ng/well) using Lipofectamine 2000 reagent (Thermo Fisher Scientific; Waltham, MA, USA) in serum-free medium (GMEM) as described previously [29]. Three hours later, the transfection mix was removed, cell monolayers were washed with GMEM and then incubated in GMEM containing or not U0126 (15 µM), and incubation proceeded for 4 h at 37 °C. Quantification of FLUC and RLUC activities were performed as described above.

### 2.14. Statistical Analysis

Statistical significance of the differences between untreated and drug-treated cultures was determined either by 2-tailed paired Student’s t test, to compare the results between two groups, or ANOVA analysis. Three independent experiments were analyzed unless stated otherwise. A *p* value < 0.05 was considered to be statistically significant.

## 3. Results

### 3.1. Effect of U0126 on Initial Steps of Viral Replicative Cycle

**Figure 1:**
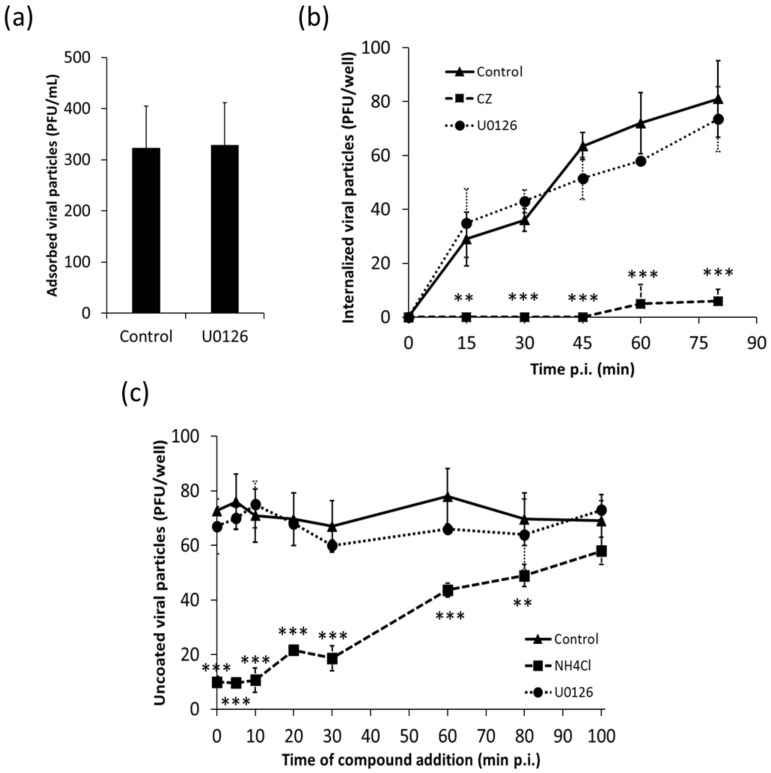
Inhibition of the ERK1/2 pathway does not affect Junín virus (JUNV) entry or uncoating. (**a**) Vero cells were mock-treated or treated with U0126 (15 µM) for 1 h, and then cells were infected with JUNV (m.o.i. = 1) in presence or absence of U0126 for 1 h at 4 °C. Following extensive washing, cells were lysed and viral infectivity was quantified by plaque assay. Data are mean values ± standard deviation (SD); (**b**) Vero cells were mock-treated or treated with U0126 (15 µM) or CZ (40 µM) for 30 min and then infected with JUNV for 1 h at 4 °C. Next, cultures were incubated at 37 °C in the presence or absence of the inhibitors. At different time points, internalized virions were quantified by plaque assay. Data are mean values ± SD. ANOVA (Tukey post-hoc test): ** *p* < 0.01, *** *p* < 0.001; (**c**) Vero cells infected with JUNV for 1 h at 4 °C were incubated at 37 °C and at the indicated time points U0126 (15 µM) or NH_4_Cl (50 mM) was added. After 3 h of treatment, uncoated viral particles were quantified by plaque assay. Data are mean values ± SD. ANOVA (Tukey post-hoc test): ** *p* < 0.01, *** *p* < 0.001.

Clade B New World (NW) arenaviruses, including JUNV and TCRV, can use transferrin receptor to bind to the cell surface allowing viral internalization through an endocytic pathway. Once viral particles are internalized, the acidic pH in endosomal compartments triggers the fusion between viral and endosomal membranes leading to the release of viral nucleocapsids into the cytoplasm [2,32]. Since we have previously proved that JUNV infection induces an early activation of the ERK pathway [23] we wondered whether MAPKs may be involved in the initial events of viral multiplication cycle. Therefore, we first analyzed the effect of the MEK inhibitor U0126 on JUNV adsorption. As shown in Figure 1a, the presence of U0126 did not affect the amount of JUNV infectious particles adsorbed to Vero cells after 1 h incubation at 4 °C. To investigate the effect of the inhibitor on viral internalization, after JUNV adsorption, cultures were incubated at 37 °C for different time periods in presence or absence of U0126 or chlorpromazine (CZ), an inhibitor of clathrin-mediated endocytosis. Whereas CZ blocked JUNV endocytic uptake, no significant differences in the kinetics of JUNV internalization were detected in U0126 treated cultures with respect to untreated ones (Figure 1b). In order to assess whether inhibition of MEK affects viral uncoating, JUNV was adsorbed at 4 °C and then cells were transferred to 37 °C. At different time points, either U0126 or NH_4_Cl, compounds that raise endosomal pH, thereby inhibiting the release of viral nucleocapsids, was added and maintained during 3 h, and uncoated viral particles were then quantified by plaque assay. As expected, NH_4_Cl significantly inhibits JUNV uncoating even when added at 80 min post-adsorption. On the contrary, the uncoating process was not affected by U0126 treatment (Figure 1c).

### 3.2. Effect of U0126 on the Expression of JUNV Proteins

**Figure 2:**
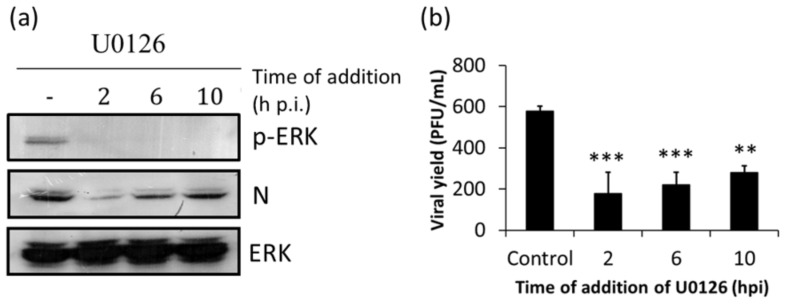
U0126 inhibits N protein expression and viral production. Vero cells were infected with JUNV (m.o.i. = 1) and at different time points U0126 (15 µM) was added. At 14 h p.i. cells were lysed and the levels of expression of N and p-ERK were analyzed by Western blot using ERK as loading control (**a**), and virus production was determined by plaque assay (**b**). Data are mean values ± SD. ANOVA (Dunnett post-hoc test): ** *p* < 0.01, *** *p* < 0.001.

To further uncover the viral step/s at which ERK1/2 MAPKs are engaged, we first analyzed the effect of the time of U0126 addition on the expression of N protein. Infected cells were examined at 14 h p.i. in order to detect the level of viral protein accumulated along one cycle of viral multiplication. Lack of cytotoxicity of U0126 treatment was assessed by the MTT method (Figure S1). Addition of the compound at 2 h p.i was associated with highly reduced levels of N expression. Likewise, a marked reduction in the amount of N was detected after addition of the inhibitor at 6 h p.i. (Figure 2a). Consistently, cells treated with U0126 from 2, 6 or 10 h p.i., exhibited a significant reduction in extracellular virus yields with respect to untreated cells, with maximum inhibition also observed after addition of the compound at 2 h p.i (Figure 2b). These results, which correlated with undetectable amounts of phosphorylated ERK (Figure 2a, upper panel), indicate that ERK1/2 MAPKs are involved in a post-entry step affecting N expression and virus production.

**Figure 3:**
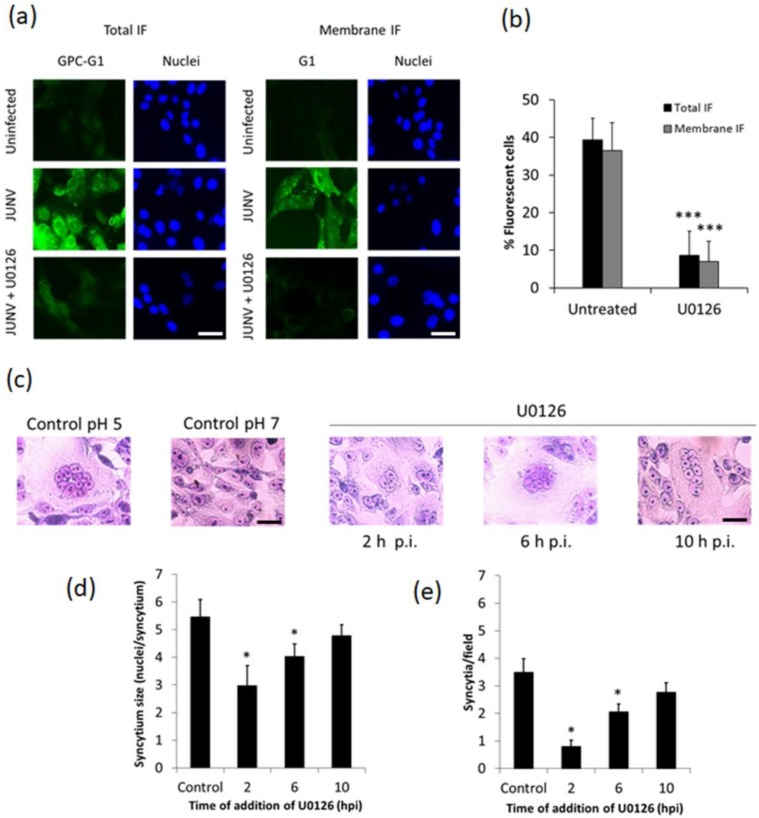
U0126 inhibits viral glycoprotein expression. (**a**) Cells were infected with JUNV (m.o.i. = 1) and U0126 (15 µM) was added 2 h later. At 24 h p.i. total and membrane G1 expression was analyzed by immunofluorescence (IF) assay. Scale bars: 10 µm; (**b**) The percentage of fluorescent cells was calculated by counting 20 random selected fields; data represent mean values ± SD. ANOVA (Tukey post-hoc test): *** *p* < 0.001; (**c**) Vero cells were infected with JUNV (m.o.i. = 1) and U0126 (15 µM) was added at the indicated time points. At 14 h p.i. cells were treated for 30 min with culture medium at pH 5.0. Ten hours later, cells were fixed, stained with Giemsa and visualized using an optic microscope. Scale bars: 10 µm; The size (**d**) and number (**e**) of syncytia were calculated by counting 20 random selected fields (400× magnification); data represent mean values ± SD. ANOVA (Dunnett post-hoc test): * *p* < 0.05.

Next, we sought to evaluate the impact of MAPK activation on the expression and function of viral glycoproteins. GPC, the glycoprotein precursor, is cleaved by host proteases to produce a stable signal peptide (SSP), the receptor binding G1 and the transmembrane G2 subunit, which mediates the low pH-dependent fusion between the viral envelope and the endosomal membrane [33]. To analyze the effect of U0126 on both cytoplasmic and plasma membrane expression of viral glycoproteins, surface and total immunofluorescence assays were performed. As can be seen in Figure 3a,b, U0126 similarly reduced the percentage of cells exhibiting either total or membrane fluorescence, indicating that the compound mainly affects the synthesis of JUNV glycoproteins rather than glycoprotein transport to the cell surface. Accordingly, when the ability of the glycoprotein complex to mediate low pH membrane fusion was evaluated in a well-established cell-cell fusion test [34], a significant decrease in the number and size of syncytia was observed in U0126-treated cells as compared with untreated cultures (Figure 3c–e). Overall, these results indicate that ERK1/2 MAPKs are required for the expression of both the nucleoprotein and the viral envelope glycoproteins.

### 3.3. Effect of U0126 on Viral RNA Synthesis

**Figure 4:**
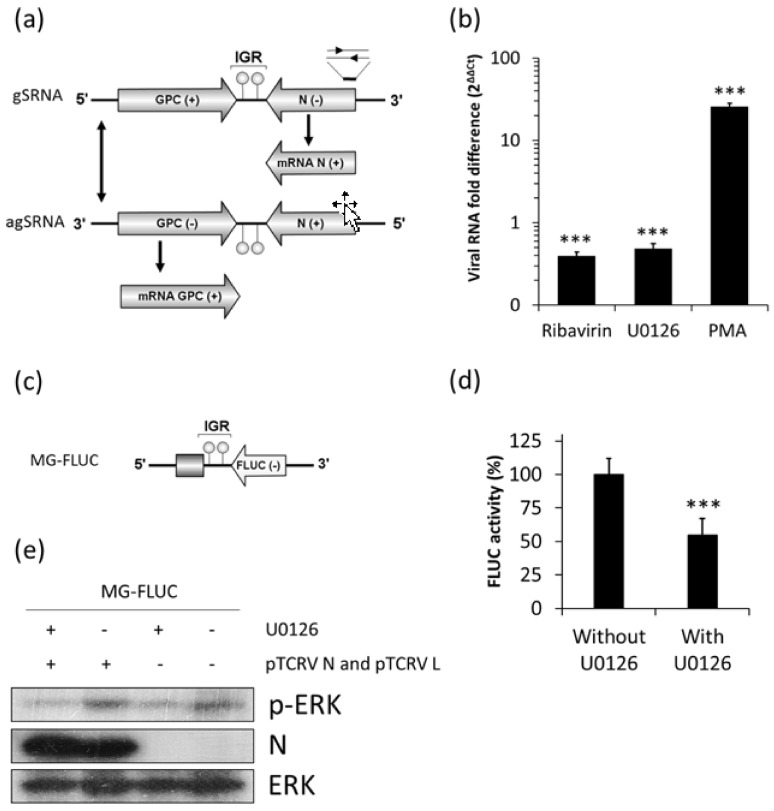
U0126 inhibits viral RNA synthesis. (**a**) Schematic representation of the S RNA transcription/replication processes. The genome S RNA (gSRNA) and the full-length antigenome S RNA (agSRNA), N mRNA, glycoprotein precursor (GPC) mRNA are depicted. IGR: intergenic region. The primers used for detection of gSRNA by Q RT-PCR are indicated with arrows; (**b**) Total RNA was extracted at 14 h p.i. from JUNV infected Vero cells treated with U0126 (15 µM), ribavirin (100 µM), PMA (100 nM), or left untreated, and gSRNA was detected by Q RT-PCR. Data represent mean values ± SD. ANOVA (Dunnett post-hoc test): *** *p* < 0.001; (**c**) Schematic representation of Tacaribe virus (TCRV) S RNA analog MG-FLUC (MG- firefly luciferase), which contains (5′ to 3′) the S genome 5′ UTR, followed by a linker sequence corresponding to the 3′ terminal region of the GPC open reading frame (ORF) (shadowed box); the complete S IGR, then the FLUC ORF in an antisense orientation, and the complete S genome 3′ UTR; (**d**) BSR cells were plasmid-transfected to co-express TCRV N and L proteins along with the MG-FLUC RNA. Transfection mixes, which included a plasmid expressing RLUC to control for transfection efficiency, were removed after 4 h and cells were further incubated in the presence of U0126. Cytoplasmic extracts, obtained at 20 h post-transfection, were assayed for FLUC and RLUC activities and FLUC activity data were normalized against the corresponding RLUC levels. Mean normalized FLUC values ± SD from two independent experiments (each performed in triplicate) are shown as a percentage of data corresponding to untreated cultures taken as 100%. 2-tailed paired Student’s *t* test: *** *p* < 0.001; (**e**) Western blot analysis of N protein and p-ERK levels in samples obtained from BSR cells transfected with MG-FLUC, expressing or not N and L in the presence or absence of U0126, as described in d. ERK detection was used as loading control.

We next investigated the effect of U0126 treatment on JUNV RNA synthesis by performing a quantitative RT-PCR assay to detect genomic S RNA (Figure 4a,b). A significant reduction in the amount of viral RNA was evident after treatment of JUNV-infected cells with U0126, comparable to the effect caused by ribavirin, used as a reference RNA synthesis inhibitor. By contrast, treatment with PMA, a known activator of the ERK1/2 pathway, which has been proved to promote JUNV production [23], strongly increased JUNV RNA synthesis as compared to untreated control (Figure 4b).

Because we have previously demonstrated that U0126 not only affects JUNV productive infection but also inhibits the multiplication of the non-pathogenic NW arenaviruses TCRV and PICV [23], we used a TCRV replicon system [28] to further assess the relevance of the ERK pathway activation on the synthesis of arenavirus RNA. In this system, co-expression of the nucleoprotein N and the viral RNA polymerase L is sufficient to promote full-cycle RNA replication of TCRV S segment analogs. Thus, we investigated the action of the MEK inhibitor on the expression of a TCRV S genome analog (MG-FLUC), containing the FLUC reporter gene and the complete TCRV S RNA noncoding sequences (Figure 4c). After treatment with U0126, FLUC expression was significantly reduced with respect to untreated cultures (Figure 4d). Importantly, the blockade of the ERK1/2 pathway did not alter N protein expression levels in transfected cells (Figure 4e) indicating that protein expression from plasmids that constitute the replicon system was not affected by the presence of the MEK inhibitor. In addition, the lack of cytotoxicity of U0126 treatment in transfected BSR cells was also corroborated by the MTT method (Figure S2). Altogether our results indicate that U0126 impairs the reporter gene expression from a TCRV S genome analog, further supporting that MAPKs display an important role in viral RNA synthesis. 

### 3.4. Effect of U0126 on Viral mRNA Translation

**Figure 5:**
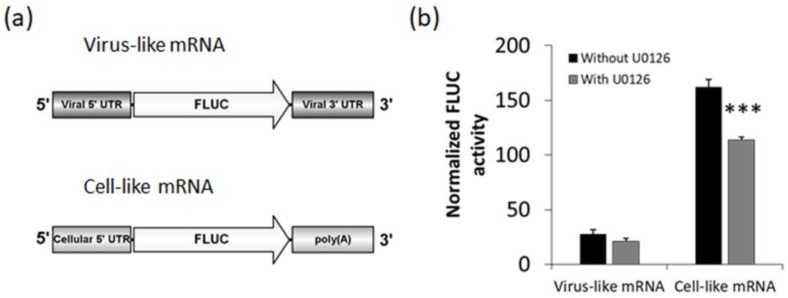
U0126 does not affect viral mRNA translation. (**a**) Schematic representation of capped synthetic transcripts used in this study; (**b**) BSR cells were transfected with virus- or cell-like mRNA, along with a transcript (RLUC), expressing RLUC in a cap-independent manner, as an internal control (Materials and methods). Three h post-transfection, cells were treated or not with U0126 for 4 h, followed by the determination of FLUC and RLUC activities in cell lysates. Mean normalized FLUC values (FLUC/RLUC ± SD) from two independent experiments (each performed in triplicate) are shown. ANOVA (Tukey post-hoc test): *** *p* < 0.001.

The reduced levels of FLUC activity observed in the context of the replicon system in U0126 treated cultures could be ascribed to a blockade of RNA synthesis, to an inefficient translation of FLUC mRNA or both. In order to distinguish between these possibilities, we performed a cell-based translation assay to analyze the effect of the ERK1/2 pathway inhibition on translation of capped synthetic transcripts. We used a virus-like mRNA that mimics the TCRV N mRNA and comprises the FLUC ORF flanked by the viral 5′ and 3′ UTRs (Figure 5a) [29]. As a control, a cell-like mRNA (β-Glo/poly(A)) [29], which bears the 5′ UTR from human β-globin mRNA, and a 53-nt 3′ poly(A) tail flanking the FLUC coding sequence, was also included in these experiments (Figure 5a). 

Translation from the virus- or cell-like transcript was examined after transfection into BSR cells followed by treatment with U0126. Whereas expression of the cell-like mRNA was slightly decreased, virus-like mRNA translation levels in U0126 treated cultures did not significantly differ from those in untreated cells (Figure 5b), suggesting that U0126-mediated inhibition of viral RNA synthesis from JUNV genome (Figure 4b), or of FLUC expression from the S genome analog (Figure 4d) did not rely on an adverse effect of the compound on viral mRNA translation.

**Figure 6:**
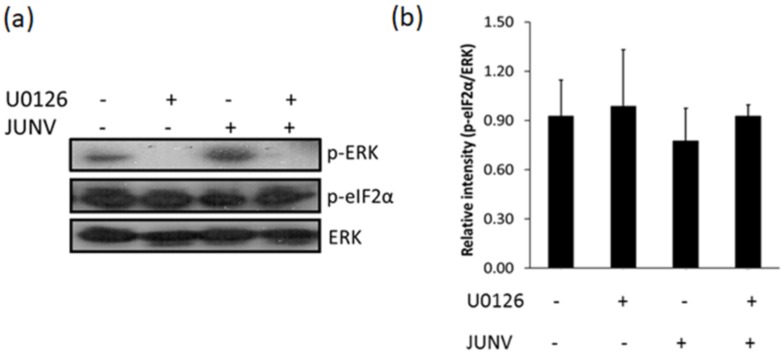
U0126 does not affect eukaryotic initiation factor 2α (eIF2α) phosphorylation. (**a**) Uninfected and JUNV infected (m.o.i. = 1) Vero cells were treated with U0126 during 12 h or were left untreated. Then cells were lysed and the level of eIF2α and ERK phosphorylation was assessed by Western blot; (**b**) Quantification of p-eIF2α, data represent mean values of relative intensity with respect to ERK ± SD.

The eukaryotic initiation factor 2α (eIF2α) is a cell factor essential for the initiation step of protein translation, which is active in its hypophosphorylated state. It has been described that MEK1/2 inhibitors, such as U0126, may promote eIF2α phosphorylation thereby inhibiting the cell translation process [35]. Thus, to analyze whether ERK1/2 inhibition could be linked to increased phosphorylation of eIF2α, we sought to quantify the levels of phosphorylated eIF2α upon treatment of JUNV infected Vero cells with U0126 for 12 h. In accordance with data previously reported [36], in the absence of U0126, the levels of eIF2α phosphorylation in JUNV-infected cells were comparable to those in uninfected cultures, as determined by Western blotting analysis. In addition, the inhibition of the ERK1/2 pathway did not induce changes in the amount of phosphorylated eIF2α (p-eIF2α) either in uninfected or in JUNV infected cells (Figure 6). These results support that the inhibition of viral protein expression and RNA synthesis observed in U0126 treated cultures would not be related to increased phosphorylation of eIF2α in host cells.

## 4. Discussion

Although it has been well established that ERK1/2 signaling is involved in the multiplication of several animal viruses much remains to be understood about the specific role that this transduction cascade plays in the viral replicative cycle. In the present study, we analyzed the inhibitory effect of U0126, an inhibitor of MEK, a component of the ERK1/2 signaling pathway, on early events of JUNV multiplication. We proved that early activation of ERK signaling is not required for JUNV adsorption, internalization or uncoating (Figure 1). In contrast, treatment of infected cells with U0126 caused a marked suppression of viral protein and RNA production (Figure 2a, Figure 3 and Figure 4b). On the other hand, JUNV RNA synthesis was significantly augmented in the presence of an activator of the ERK1/2 pathway (Figure 4b). These findings were strongly supported by the analysis of the effect of U0126 on a TCRV-based reverse genetic system (Figure 4d). A cell-based translation assay revealed that translation of a TCRV-like mRNA remains unaffected in the presence of U0126 (Figure 5b), providing further evidence that the inhibitor would impair viral RNA synthesis thereby leading to a subsequent reduction in viral protein expression. Despite the lack of significant inhibitory effect of U0126 on virus-like mRNA translation, translation of the cell-like mRNA appeared to be moderately reduced in cells treated with the MEK inhibitor (Figure 5b). However, this minor adverse effect would not seem to be relevant for cell translation efficiency. Indeed, the levels of viral proteins expressed in the context of the replicon system, or ERK levels in infected cells, were unaffected by the MEK inhibitor (Figure 4e and Figure 6a). ERK1/2 pathway not only regulates cellular gene transcription but also exerts a post-transcriptional control of certain mRNAs, such as AU-rich element (ARE) containing mRNAs, which encode for a variety of proteins involved in cell proliferation and inflammatory/immune responses [37,38]. Therefore, the possibility that U0126 may downregulate the translation of a subset of cellular mRNAs without affecting the expression of other transcripts, including the N protein transcript, cannot be ruled out. Moreover, we demonstrated that U0126 treatment did not promote an increment in the amount of phosphorylated eIF2α, regardless of virus infection (Figure 6). In summary, our data reveal that the U0126-induced inhibition is independent of eIF2α phosphorylation and that it is not linked to altered host translation levels, further supporting that ERK1/2 MAPKs play an important role in viral RNA synthesis.

In agreement with our findings, Cai et al. [39] showed that U0126 hinders the synthesis of mouse hepatitis virus (MHV) genomic and subgenomic RNAs, whereas viral entry or translation of MHV mRNAs appeared to be unaffected by the MEK inhibitor.

A great deal of our knowledge about ERK1/2 pathway participation in the replication of RNA viruses comes from studies on influenza A (IVA) and B (IVB) viruses [15,17,40,41]. The lack of effect of U0126 on early steps of JUNV multiplication is in agreement with data obtained by Marjuki et al. [41] in IVA infected cells. However, in contrast with our results, MEK inhibition did not affect the synthesis of IVA and IVB RNA but impaired the exportation of newly synthesized viral ribonucleoproteins (RNPs) from the cell nucleus to the cytoplasm hindering viral assembly [15,17]. Even though ERK-mediated phosphorylation of NS1 IVA protein has been reported [42] the requirement of NS1 phosphorylation for RNP transport from the nucleus to the cytoplasm has not been demonstrated. In addition, it has been proposed that a host cell factor, regulated by ERK phosphorylation, might be involved in the exportation of IV RNPs [17]. Analysis of JUNV N amino acid sequence, using PhosphoMotif Finder program, revealed that JUNV nucleoprotein contains three potential ERK1/2-mediated phosphorylation motifs. It was previously demonstrated that self-association between N protein molecules, as well as the interaction between N and L proteins, are required to sustain transcription and replication of TCRV MG RNA [26,43]. Therefore, either direct phosphorylation by ERK or ERK-regulated phosphorylation of N might be relevant for the formation and/or activity of viral protein complexes that carry out viral RNA synthesis. Hence, blocking ERK signaling might hinder N phosphorylation thereby inhibiting JUNV genome replication. Indeed, two residues in the nucleoprotein of the arenavirus lymphocytic choriomeningitis virus (LCMV), which are conserved in every mammalian arenavirus, were demonstrated to be required for recombinant LCMV recovery, suggesting their involvement in regulation of viral RNA replication [44]. Besides the requirement of a coordinated interaction between N, L and viral RNA, arenavirus RNA synthesis likely involves interactions with host cell factors [45]. Prohibitin (PHB), one of the upstream components of the ERK1/2 pathway, has been proved to interact with JUNV and LCMV N protein [46] and it has been recently proved that a PHB inhibitor, rocaglamide, impaired LCMV RNA synthesis [45]. Moreover, JUNV multiplication was also inhibited by rocaglamide providing further evidence of the relevance of the ERK1/2 pathway activation on arenavirus RNA synthesis [45]. Thus, yet unknown cellular proteins, possibly as part of viral transcriptase/replicase complexes may be targets of or regulated by ERK1/2-mediated phosphorylation.

We found evidence that the presence of the MEK inhibitor from 2 to 14 h p.i. caused a 50% decrease in viral RNA synthesis (Figure 4b), and a 69% inhibition of virus production (Figure 2b). On the other hand, treatment with U0126 from 10 to 14 h p.i. had little effect on N protein expression (Figure 2a) but caused around 60% reduction on virus yield (Figure 2b). Interestingly, inhibition of PHB by rocaglamide not only affects LCMV RNA synthesis but also budding of LCMV virus-like particles [45]. On this regard, the possibility that ERK1/2 pathway may be engaged in late steps of virus multiplication cycle, such as budding or release of viral particles, cannot be discarded and remains to be assessed. 

Recent advances in the knowledge of arenavirus biology have greatly contributed to the discovery of novel antiviral strategies [2]. ERK1/2 modulation by arenaviruses makes this signaling pathway an attractive target for antiviral intervention and represents a strategy with low risk of viral resistance appearance [15], which could be effective against different arenaviruses. Moreover, antiviral cellular targets offer the possibility of repurposing licensed compounds that have been approved to treat other human diseases. It is promising that MEK inhibitors with known safety-data profiles are actually being under evaluation in cancer clinical trials [22,47]. 

The experiments performed in the present study in order to identify the step of the JUNV and TCRV replication cycles at which U0126 exerts its inhibitory action indicate that ERK1/2 signaling is important for efficient viral RNA synthesis. Further investigations to assess whether ERK activation induces changes in the phosphorylation state of either viral or cellular factors will help to identify cellular ERK targets involved in viral RNA production.

## Supplementary Materials

The following are available online at http://www.mdpi.com/1999-4915/10/4/199/s1, Figure S1: Effect of U0126 treatment on the viability of JUNV infected Vero cells; Figure S2: Effect of U0126 treatment on the viability of BSR cells.
